# The diagnosis and treatment of septic hip with osteonecrosis of the femoral head

**DOI:** 10.1186/s13018-023-04518-6

**Published:** 2024-01-09

**Authors:** MingYang Li, ZhenShuai Shao, HaoXiang Zhu, YongTao Zhang

**Affiliations:** Department of Joint Surgery, The Affiliated Hospital of Qingdao University, Qingdao, 266000 Shandong China

**Keywords:** Osteonecrosis of femoral head (ONFH), Septic hip, Diagnosis, Two-stage arthroplasty

## Abstract

This article aims to provide clinical doctors with references for the diagnosis and treatment of osteonecrosis of the femoral head (ONFH) accompanied with septic hip by summarizing and analyzing clinical data and postoperative follow-up information of patients treated with two-stage arthroplasty. We retrospectively analyzed ten patients who underwent two-stage arthroplasty in our hospital due to ONFH accompanied with septic hip. The diagnosis of septic hip includes erythrocyte sedimentation rate (ESR) > 30 mm/h, C-reactive protein (CRP) > 10 mg/L, pus-like synovial fluid, positive microbiological culture, and the findings of septic arthritis on magnetic resonance imaging (MRI) scan. Patient's information was evaluated based on the review of medical records, including gender, age, symptoms, risk factor of ONFH and septic arthritis, blood test, radiograph, MRI scan, microbiological culture, treatment, follow-up period and outcome. A total of ten patients were diagnosed with ONFH accompanied with septic hip. The average follow-up period was 43.5 months. None of the patients experienced failure during the follow-up period. The risk factor of ONFH was alcohol-related (60%), steroid-related (20%) and idiopathic (20%). Nine patients (90%) have no risk factor of septic arthritis and one patient (10%) has nephrotic syndrome. All patients did not experience any fever symptoms before surgery, but all showed worsening symptoms of pain. There were three patients (30%) with abnormal WBC count > 10 × 10^9^/L. All patients had elevated ESR and/or CPR. Nine patients (90%) had positive MRI findings, and seven patients (70%) had positive microbiological culture. When patients with ONFH experience worsening hip joint pain accompanied by unexplained elevated CRP and/or ESR, it should be suspected whether ONFH is accompanied with septic hip. In these cases, MRI scans should be performed to exclude septic hip. Patients with ONFH accompanied with septic hip showed satisfactory results after two-stage arthroplasty.

## Introduction

Osteonecrosis of the femoral head (ONFH) is a debilitating and potentially devastating condition that affects patients' quality of life, causing pain and dysfunction in walking [1]. It has a poorly understood pathogenesis and wide-ranging aetiologies [2]. Total hip arthroplasty (THA) is the most reliable treatment method for reducing pain and restoring mobility in end-stage of ONFH [3]. However, while improving patients' quality of life, it can also lead to the occurrence of complications [4].

Periprosthetic joint infection (PJI) is one of the most serious complications of THA [5]. Among the various treatment options, the most commonly used way in the USA and many other countries is known as two-stage arthroplasty [6–8]. It involves removing the prosthesis and all foreign materials, followed by a delayed implantation of a new prosthesis. PJI not only significantly jeopardizes the patient's well-being but also imposes a substantial financial burden on them [9, 10]. Consequently, many researchers have focused their attention on THA and its postoperative complications, but have tended to overlook a critical problem: ONFH accompanied with septic hip.

Septic hip is rare in patients with ONFH. Clinical symptoms of these patients usually are hidden and specific diagnostic indicators are scarce, so surgeons may overlook these patients [11, 12]. However, if THA is performed on patients with ONFH accompanied with septic hip, PJI is inevitable and will be a devastating disaster for the patient. To date, there have been few reports on septic hip in patients with ONFH. This article aims to provide clinical doctors with references for the diagnosis and treatment of ONFH accompanied with septic hip by summarizing and analyzing clinical data and postoperative follow-up information of patients who were treated with two-stage arthroplasty.

## Materials and method

This is a retrospective study that included 10 patients who underwent two-stage arthroplasty in our hospital from July 2015 to October 2021 due to ONFH accompanied with septic hip. After admission screening, all patients had no other site infections. This study has been approved by the institutional review board and obtained written informed consent from all patients. Two senior surgeons performed two-stage arthroplasty for 10 patients. After admission, blood tests, ESR, and CRP were collected from all patients. Prior to the surgery, X-ray and magnetic resonance imaging (MRI) scans were conducted. Patients' information was evaluated based on the review of medical records, including gender, age, symptoms, risk factors of ONFH and septic arthritis.

**Fig. 1:**
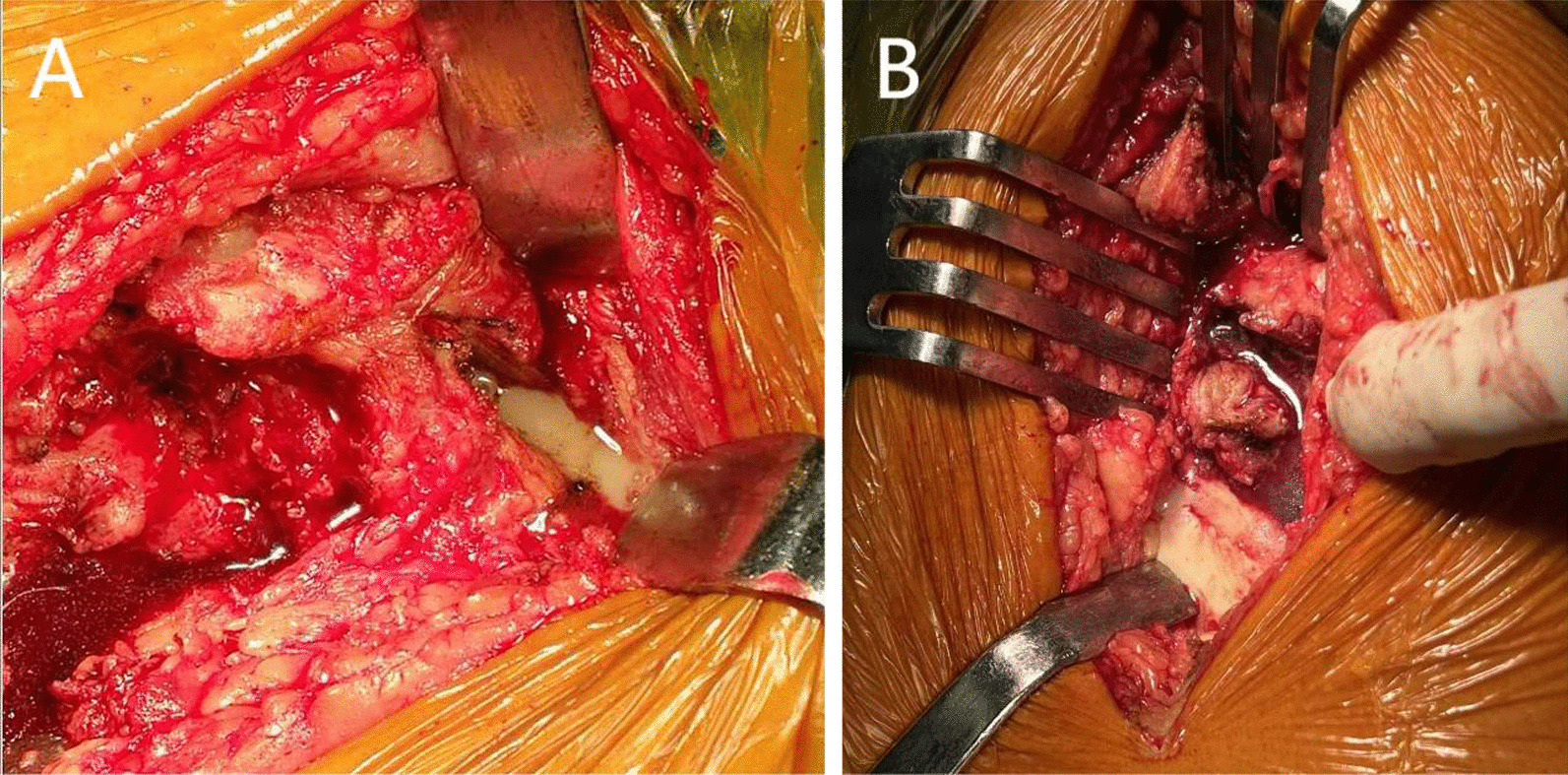
During the surgery, pus-like fluid gushed out from the hip joint

**Fig. 2:**
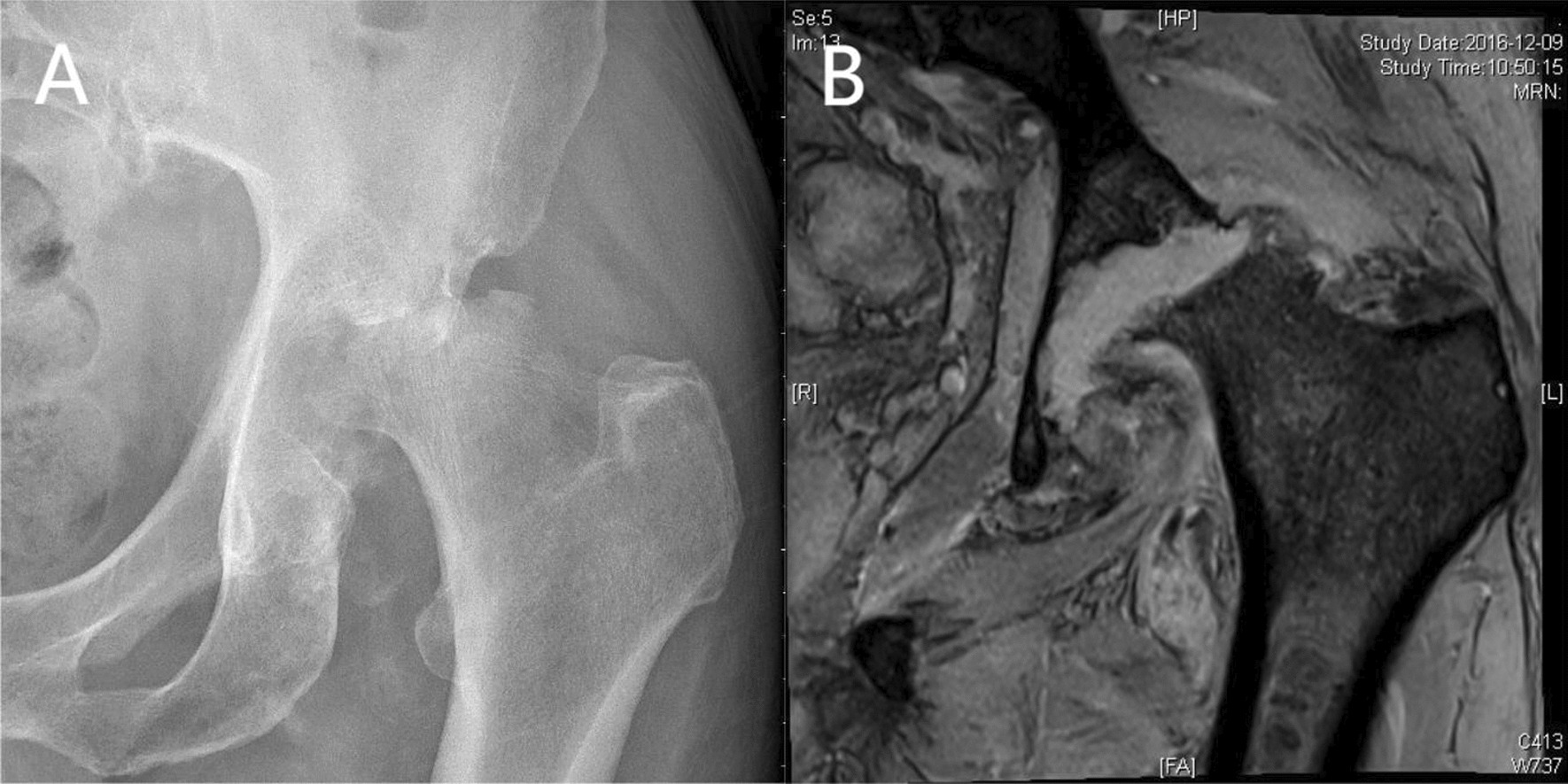
A 67-year-old male patient suffered from ONFH accompanied with septic hip (case 6). **A** The initial radiograph revealing the collapse of femoral head and narrowing of joint space. **B** MRI shows a large amount of effusion and synovial thickening of the hip

In this study, the diagnosis of septic hip includes ESR > 30 mm/h, CRP > 10 mg/L, pus-like synovial fluid (Fig. 1), positive microbiological culture, the findings of septic arthritis on MRI scans (Fig. 2). MRI findings that were considered as positive for septic arthritis included an increase of joint effusion, presence of synovial thickening, alterations in signal intensity of bone marrow of both proximal femur and acetabulum and soft tissue around the hip joint with strong enhancement [13].

**Fig. 3:**
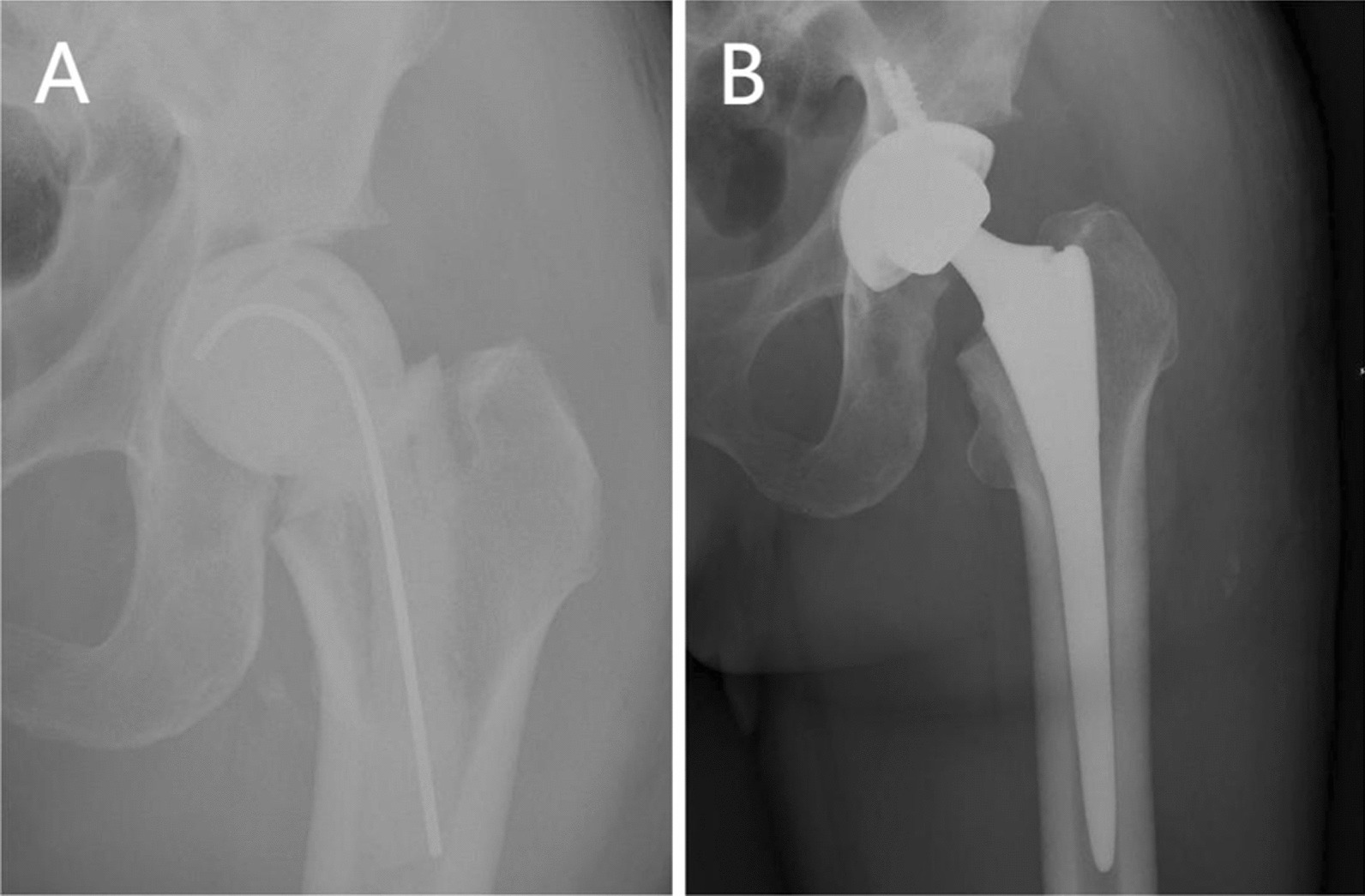
Standard two-stage arthroplasty (case 6). **A** Radiograph after an antibiotic-loaded cement spacer inserted. **B** Radiograph after two-stage arthroplasty

All patients underwent a standard two-stage arthroplasty (Fig. 3). The first stage includes removal of the femoral head, and aspiration of synovial fluid for pathogenic culture. At least three surrounding deep tissues were sent for histological analysis and a radical debridement was performed on the affected joint. An antibiotic-loaded articular bone cement spacer, containing 2 g of vancomycin and 0.5 g of gentamicin per 40 g bone cement (Refobacin® Bone Cement R, Zimmer Biomet), was then inserted. Patients received 2 weeks of intravenous antibiotics plus 4 weeks of oral antibiotics. Antibiotics were selected and used according to the drug susceptibility results of pathogen culture and the opinions of infectious disease specialists. Regular monitoring of inflammatory markers was conducted during antibiotic treatment. Afterwards, use of antibiotics was suspended for at least 6 weeks. Patients were regularly tested for clinical and inflammatory indicators, and if necessary, aspiration of synovial fluid was performed for culture. Reimplantation will not be considered feasible until the patient has no clinical signs of infection, the surgical wound has healed up, and the ESR and CRP are gradually decreasing. The second stage included removal of the cement spacer, aspiration of synovial fluid for pathogenic culture, sending periprosthetic tissues for histological analyses, debridement and irrigation once again, and then implant the prosthesis. Intravenous antibiotics were routinely administered postoperatively, and drug was discontinued when the intraoperative culture results turned negative. After discharge, patients were routinely followed up in outpatient clinics, and ESR, CRP and X-ray were performed according to each patient's condition. When patients need revision surgery for various reasons, it is considered a failure of the surgery.

The classification schemes for ONFH of Ficat were evaluated twice, once by the specially trained surgeon and once by a blinded surgeon [14]. The intraclass correlation coefficient was measured to be > 0.8, indicating good reproducibility.

## Results

Previous studies included a total of 34 patients with ONFH accompanied with septic hip (Table 1). In this study, a total of ten patients (six males and four females) were diagnosed with ONFH accompanied with septic hip. According to the Ficat staging criteria for ONFH, nine patients (90%) were in Ficat stage IV and one patient (10%) was in Ficat stage III. The average age was 67.5 years (55–76), and the average follow-up period was 43.5 months (22–91). None of the patients experienced any failure during the follow-up period. The risk factor of ONFH were alcohol-related (60%), steroid-related (20%) and idiopathic (20%). Nine patients (90%) have no risk factor of septic arthritis and one patient (10%) has nephrotic syndrome. All patients did not experience any fever symptoms before surgery, but all showed worsening symptoms of hip joint pain. There were three patients (30%) with an abnormal WBC count > 10 × 10^9^/L, eight patients (80%) with ESR > 30 mm/L, and nine patients (90%) with CPR > 10 mg/L. All patients had elevated ESR and/or CPR. Nine patients (90%) had positive MRI findings, and seven patients (70%) had positive microbiological culture (Table 2).

**Table 1:** Previously reported 34 patients with ONFH accompanied with septic hip

Study	Sex/age	Risk factor of ONFH	Risk factor of Septic arthritis	Fever	WBC count (% of PMN)	ESR (mm/h)	CRP (> 0.5 mg/dL)	WBC count of joint fluid (% of PMN)	MRI (infection)	Culture of joint fluid	Treatment
Galindo et al. (2005)	F/47	CS with SLE	CS with SLE	+	/	/	/	/	+	MSSA	Antibiotics
	F^ab^/38	CS with SLE	CS with SLE	+	/	/	/	/	+	MSSA	Arthrotomy
									+	MSSA	Arthrotomy
Ostrum et al. (1993)	F/51	CS for heart TPL	Norcadia pneumonia	+	10,400 (90%)	/	/	/	/	*Norcardia*^a^	Arthrotomy
Nuovo et al. (1991)	M^c^/45	Alcohol-related	Intravenous drug abuse	−	4,000 (47%)	36	/	/	/	*S. aureus*^c^	Arthrotomy
Philips et al. (1988)	M^a^/32	CS for HD	Subacute endocarditis	+	12,800	67	/	/	/	*S. viridans*	Arthrotomy
	M^a^/53	Idiopathic	None	−	6,300	95	/	/	/	*S. aureus*	Arthrotomy
	M/14	Sickel cell disease	Pharyngitis	+	17,700	/	/	20,000	/	*H. influenza*	Arthrotomy
	M^a^/33	CS for renal TPL	Pharyngitis	−	18,700	/	/	/	/	*Streptococcus*	Arthrotomy
Shiota et al. (1981)	F/36	CS with SLE	GI Salmonellosis	+	12,500	127	/	/	/	*Salmonella typhimurium*^*c*^	Arthrotomy
Habermann et al. (1978)	F/25	CS for renal TPL	Deep infection after TPL	+	/	/	/	/	/	*S. aureus*^*c*^	Repeated aspiration
	F/21	CS with SLE	Skin infection	+	/	/	/	/	/	Negative^c^	Repeated aspiration
	M^ab^/30	CS for renal TPL	Salmonella sepsis	+	/	/	/	70,000	/	Negative^c^	Repeated aspiration
								30,000	/	*Salmonella*^c^	Repeated aspiration
	M/20	CS for renal TPL	None	+	/	/	/	24,000	/	*Peptostreptococcus*	Repeated aspiration
Lee et al. (2011)	M/36	Alcohol-related	None	−	4,280 (49%)	29	3.35	110,000 (89%)	/	Negative	Arthroscopy
	M^a^/75	Alcohol-related	None	−	7,260 (72%)	53	3.15	/	+	Negative	Arthroscopy
	M^a^/48	Alcohol-related	None	−	6,120 (63%)	19	5.39	/	/	Negative	Arthrotomy
	M^a^/51	Alcohol-related	Osteomyelitis of toe	+	12,770 (46%)	18	1.13	23,000 (86%)	+	Negative^c^	Arthroscopy
	M^a^/37	Steroid-related	Subcutaneous abscess	+	7,090 (68%)	23	4.12	195,000 (96%)	+	MRCNS^c^	Arthroscopy
Kim et al. (2018)	M/50	Alcohol-related	alcoholic liver cirrhosis end-stage renal disease	+	13,100	76	7.21	/	+	*Stenotrophomonas maltophilia*^*c*^	Arthroscopy
	F/44	Idiopathic	None	+	/	62	14.7	400,000 (73%)	+	*S. aureus*^c^	Arthroscopy
Lee et al. (2019)	14 patients	/	None	3 patients+	/	All patients had elevated serum ESR (> 20 mm/h) and/or CRP (> 0.5 mg/dL)		/	12 patients+	/	Two-stage THA

*ONFH* Osteonecrosis of the femoral head, *PMN* polymorphoneutrophil, *ESR* erythrocyte sedimentation rate, *CRP* C-reactive protein, *CS* corticosteroid, *SLE* systemic lupus erythematosus, *MSSA* methicilin-sensitive *Staphylococcus aureus*, *TPL* transplantation, *HD* Hodgkin disease, / Not recorded

^a^Bilateral *ONFH*

^b^Bilateral septic arthritis

^c^Previous use of antibiotics

**Table 2:** Patients with ONFH accompanied with septic hip in this study

	Sex/age	Risk factor of ONFH	Risk factor of Septic arthritis	Fever	WBC count (% of PMN)	ESR	CRP	WBC count of joint fluid (% of PMN)	MRI (infection)	Culture of joint fluid	Treatment	Follow-up period (mo)	Outcome
Patient 1	F/67	Alcohol-related	None	−	5.26(83.50)	39.7	52.25	/	+	*Corynebacterium*	Two-stage reimplantation	28	No Failure
Patient 2	M/76	Alcohol-related	None	−	6.8(58.10)	19.5	51.69	/	+	Negative	Two-stage reimplantation	32	No Failure
Patient 3	F/60	Alcohol-related	None	−	9.86(69.30)	29.4	9.86	/	+	MSSA	Two-stage reimplantation	22	No Failure
Patient 4	M/55	Alcohol-related	None	−	8.93(86.50)	50.6	154.33	/	+	MSSA	Two-stage reimplantation	26	No Failure
Patient 5	F/81	Idiopathic	None	−	7.04(57.90)	56.3	30.3	/	-	Negative	Two-stage reimplantation	25	No Failure
Patient 6	M/67	Alcohol-related	None	−	7.67(62.50)	58	31.72	/	+	*Salmonella*	Two-stage reimplantation	78	No Failure
Patient 7	M/74	Alcohol-related	None	−	17.29(83.80)	202	48.6	/	+	*Citrobacter*	Two-stage reimplantation	65	No Failure
Patient 8	M/68	Steroid-related	None	−	16.5(68.14)	42.3	46.46	/	+	Negative	Two-stage reimplantation	91	No Failure
Patient 9	F/70	Steroid-related	nephrotic syndrome	−	8.39 (78.30)	114	165	/	+	*Salmonella*	Two-stage reimplantation	45	No Failure
Patient 10	M/64	Idiopathic	None	−	13.41(81.1)	102	194.77	/	+	*Klebsiella*	Two-stage reimplantation	23	No Failure

*ONFH* Osteonecrosis of the femoral head, *PMN* polymorphoneutrophil, *ESR* erythrocyte sedimentation rate, *CRP* C-reactive protein, *MSSA* methicilin-sensitive *Staphylococcus aureus*,

+positive, − negative, / Not recorded

## Discussion

Septic hip usually occurs in children, while in adults it is more common in patients with autoimmune diseases [15, 16]. Patients with ONFH accompanied with septic hip are rare. The possible reason is that the necrotic tissue in the femoral head provides a favorable environment for bacterial proliferation, leading to the occurrence of infections. The clinical symptoms of patients with ONFH accompanied with septic hip are usually hidden. Such patients often have a history of alcoholism, systemic lupus erythematosus (SLE), steroid therapy, and varying degrees of immune function suppression [17]. There is also a lack of specific diagnostic indicators for diagnosis. Once such patients are misdiagnosed, they are prone to PJI after undergoing THA. Therefore, it is necessary to conduct various examinations to exclude septic hip in patients with ONFH after admission.

Patients with limited immune function have a higher risk of ONFH accompanied with septic hip. Previous studies reported a total of 34 patients with ONFH accompanied with septic hip, of which 12 patients (35.3%) had limited immune function or risk factors of septic arthritis, and 22 patients (64.7%) had normal immune function [11, 12, 17–23]. In our study, only 2 patients (20%) required steroid therapy due to nephrotic syndrome, while the remaining 8 patients (80%) had normal immune function. This study shows that patients without risk factors of septic arthritis and limited immune function, as well as those with immune deficiency diseases and receiving steroid therapy, are prone to ONFH accompanied with septic hip. The possible reason is that steroid therapy and other immunosuppressants weaken the body's immune function, increasing the risk of infection for patients [24]. In addition, when the body experiences infection or inflammation, alcohol can inhibit the function of polymorphoneutrophil and their ability to clear pathogens [25, 26].

WBC, ESR, and CPR are routine tests before total joint arthroplasty and are also one of the criteria for diagnosing PJI [27, 28]. Lee and Kim et al. [11, 12, 23] reported a total of 21 patients with ONFH accompanied with septic hip, all of whom had elevated ESR and/or CPR. ESR > 30 mm/h and CRP > 10 mg/L were used as one of the diagnostic criteria in this study. Nine patients (90%) met the criterion, and although one patient (10%) did not meet this criterion, ESR and CPR were abnormally increased. Only three patients (30%) had elevated WBC. In addition, from July 2015 to October 2021, we performed THA on 4 patients with significantly elevated ESR and CPR for ONFH (Table 3). All patients had normal WBC, negative MRI findings and microbiological culture. The average follow-up period was 61.5 months (19–90), and none of them had undergone revision surgery for any reason. This study shows that WBC has poor diagnostic ability for septic hip, while ESR and CPR show high specificity. Patients with normal ESR and CPR can rule out the possibility of septic hip, while patients with elevated ESR and CPR need further examination to exclude septic hip.

**Table 3:** Patients with elevated ESR and CPR for ONFH

	Sex/age	Risk factor of ONFH	Risk factor of Septic arthritis	Fever	WBC count (% of PMN)	ESR	CRP	WBC count of joint fluid (% of PMN)	MRI (infection)	Culture of joint fluid	Treatment	Follow-up period (mo)	Outcome
Patient 1	F/71	Steroid-related	None	−	9.23 (70.00)	62.8	21.98	/	−	Negative	THA	19	No Failure
Patient 2	M/43	Alcohol-related	None	−	6.81 (70.00)	31.3	71.93	/	/	Negative	THA	90	No Failure
Patient 3	F/78	Alcohol-related	None	−	6.36 (77.84)	83	131.82	/	/	Negative	THA	81	No Failure
Patient 4	M/59	Alcohol-related	None	−	6.7 (34.40)	45	23.29	/	+	Negative	THA	56	No Failure

*ONFH* Osteonecrosis of the femoral head, *PMN* polymorphoneutrophil, *ESR* erythrocyte sedimentation rate, *CRP* C-reactive protein, + positive, − negative, / Not recorded

The first imaging modality of an infected joint should be a radiograph. Radiographic abnormalities, which include soft tissue swelling, joint space loss, periarticular osteopenia, and central or marginal osseous erosions, may be delayed following clinical onset of infection [29, 30]. In this study, nine patients (90%) were in Ficat stage IV and one patient (10%) was in Ficat stage III. Therefore, we propose a hypothesis that patients with ONFH of Ficat stage IV are more likely to be accompanied by septic hip. Other imaging modalities are more sensitive and specific than plain radiographs for detecting inflammation or effusions especially early in the disease process. MRI has high sensitivity for the diagnosis of musculoskeletal infection and also delineates with detail the extent of the osseous and soft tissue involvement [31]. Hopkins et al. [32] found that MRI with IV gadolinium had a sensitivity of 100% and specificity of 77%. The findings seen on MRI in the setting of septic arthritis include joint effusion, destruction of cartilage, and the presence of cellulitis in the soft tissues surrounding the joint in question [31]. Karchevsky et al. [13] reported that synovial enhancement, peri-synovial edema, and joint effusion had the best correlation with the presence of a septic joint. Galindo, Lee, and Kim et al. [11, 12, 18, 23] reported a total of 24 patients with ONFH accompanied with septic hip. Among them, 22 patients (91.7%) showed positive MRI findings, and 2 patients did not undergo MRI scan before surgery. In this study, 9 patients (90%) showed positive MRI findings. We believe that MRI is a valuable diagnostic tool in ONFH accompanied with septic hip. The information provided may be useful for patient management and preoperative planning.

Microbiological culture can not only detect the presence of infection but also provide information on drug sensitivity and drug resistance [33]. However, one particular problem is that surgeons may lack accessible synovial fluid, or a “dry tap,” when diagnostic aspiration is performed. Microbiological culture cannot be performed using common methods before surgery in these patients. Saline solution lavage and reaspiration for culture in patients with insufficient synovial fluid before surgery may be a sound practice [34]. However, the 2018 ICM recommended that clinicians should avoid the practice of saline lavage aspiration because it has a dilution effect on synovial fluid tests and causing artificially reduced sensitivity [35]. In this study, we did not perform synovial fluid aspiration on patients before surgery. All patients underwent intraoperative joint fluid microbiological culture, and 7 patients (70%) showed positive culture. Previous studies reported 20 patients, of which 14 patients (70%) were positive for bacterial culture. The low positive rate may be attributed to some patients having received antibiotic treatment before admission, delayed testing, and insufficient microbiological culture time.

Multiple studies have shown that two-stage arthroplasty is not only an effective method for treating PJI, but also a reliable solution for treating septic arthritis. In this study, we performed two-stage arthroplasty on 10 patients with ONFH accompanied with septic hip. During an average follow-up period of 43.5 months, all patients showed no evidence of recurrent infection and did not underwent revision surgery for any reason. In a retrospective study, Lee et al. [12] performed two-stage arthroplasty on 14 patients with ONFH accompanied with septic hip. During a follow-up period of 1–7 years, all patients showed no signs of infection. Diwanji et al. [36] performed two-stage arthroplasty on 9 patients with septic hip, with an average follow-up period of 42 months. It was found that Two-stage reconstruction using an antibiotic-loaded cement spacer was found to give satisfactory results for the treatment of hip infections with various etiologies. Additionally, ONFH accompanied with septic hip can also be treated by arthroscopic surgery. Kim et al. [23] described the technique and clinical outcome of minimally invasive arthroscopic resection arthroplasty for septic hip arthritis concomitant with ONFH. During the follow-up period, there was no recurrence of infection. One patient underwent THA at 18 months after surgery, but the postoperative condition of THA was not recorded. Lee et al. [11] performed arthroscopic surgery on 5 patients with ONFH accompanied with septic hip. During the follow-up period, there were no recurrent infections. Two patients underwent THA at 5 and 10 months postoperatively, respectively. One patient had no recurrent infections at 8 months after THA surgery, and another patient did not record the condition of THA. However, a coexistence of osteomyelitis with septic hip arthritis is regarded as a contraindication to arthroscopic treatment due to concerns of incomplete debridement of infected tissue and relapse of infection [37, 38]. In this study, patients with ONFH accompanied with septic hip showed satisfactory results after two-stage arthroplasty. However, there have been no research comparing the long-term effects of these two methods in treating ONFH accompanied with septic hip, and more research is needed to discuss in the future.

This study has some limitations. First, limited number of cases, which is related to the lower rate of ONFH accompanied with septic hip. Lee et al. [12] reported 1226 patients with ONFH, of which only 14 patients (1.1%) were accompanied with septic hip. Additionally, due to concerns about PJI, many surgeons did not perform THA on patients with elevated ESR and CRP, resulting in a lower detection rate for patients with ONFH accompanied with septic hip. Second, we could not compare the functional outcomes of both procedures because of a lack of the records regarding postoperative function scores. Finally, the MRI exams were not independently reviewed by at least two radiologists to reach a consensus on the diagnostic findings and there might be some variability in identified the extent of the infections.

## Conclusion

When patients with ONFH experience worsening hip joint pain accompanied by unexplained elevated CRP and/or ESR, it should be suspected whether ONFH is accompanied with septic hip. In these cases, MRI scans should be performed to exclude the possibility of septic hip. Due to the low positive rate of microbiological culture, we suggest treating as ONFH accompanied with septic hip when ESR and CRP significantly increase and MRI indicates infection, even if microbiological culture is negative.

## Abbreviations

ONFH: Osteonecrosis of the femoral head
ESR: Erythrocyte sedimentation rate
CRP: C-reactive protein
MRI: Magnetic resonance imaging
THA: Total hip arthroplasty
PJI: Periprosthetic joint infection
SLE: Systemic lupus erythematosus
